# 1,4-Bis(3-chloro­prop­oxy)benzene

**DOI:** 10.1107/S1600536810050348

**Published:** 2010-12-08

**Authors:** Yufei Wang

**Affiliations:** a Faculty of Science, Rm No 207, Carslow Building F07, The University of Sydney, NSW 2006, Australia

## Abstract

The mol­ecule of the title compound, C_12_H_16_Cl_2_O_2_, has a center of inversion at the centroid of the benzene ring and the asymmetric unit contains one half-mol­ecule. Inter­molecular C—H⋯π inter­actions stabilize the crystal structure.

## Related literature

For general background to the use of alk­oxy­benzene derivatives as inter­mediates in organic synthesis, see: Dudones & Pearson *et al.* (2000 ▶); Chen & Chao  (1996 ▶); Jin *et al.* (2010 ▶; Rabindranath *et al.* (2006 ▶); Zhang & Tieke (2008 ▶); Zhu *et al.* (2007 ▶). For bond-length data, see: Allen *et al.* (1987 ▶).
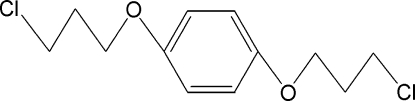


## Experimental

### 

#### Crystal data

C_12_H_16_Cl_2_O_2_
*M* *_r_* = 263.15
Monoclinic, 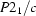

*a* = 4.9813 (8) Å
*b* = 8.3200 (14) Å
*c* = 15.273 (2) Å
β = 93.156 (6)°
*V* = 632.02 (17) Å^3^
*Z* = 2
Mo *K*α radiation
μ = 0.50 mm^−1^
*T* = 113 K
0.22 × 0.20 × 0.18 mm

#### Data collection

Rigaku Saturn diffractometer
Absorption correction: multi-scan (*CrystalClear*; Rigaku, 2000 ▶) *T*_min_ = 0.899, *T*_max_ = 0.916
5847 measured reflections
1502 independent reflections
1273 reflections with *I* > 2σ(*I*)
*R*_int_ = 0.033

#### Refinement

*R*[*F* ^2^ > 2σ(*F* ^2^)] = 0.031
*wR*(*F* ^2^) = 0.078
*S* = 1.07
1502 reflections
73 parameters
H-atom parameters constrained
Δρ_max_ = 0.37 e Å^−3^
Δρ_min_ = −0.16 e Å^−3^

### 

Data collection: *CrystalClear* (Rigaku, 2000 ▶); cell refinement: *CrystalClear*; data reduction: *CrystalClear*; program(s) used to solve structure: *SHELXS97* (Sheldrick, 2008 ▶); program(s) used to refine structure: *SHELXL97* (Sheldrick, 2008 ▶); molecular graphics: *CrystalStructure* (Rigaku, 2000 ▶); software used to prepare material for publication: *CrystalStructure*.

## Supplementary Material

Crystal structure: contains datablocks global, I. DOI: 10.1107/S1600536810050348/bq2255sup1.cif

Structure factors: contains datablocks I. DOI: 10.1107/S1600536810050348/bq2255Isup2.hkl

Additional supplementary materials:  crystallographic information; 3D view; checkCIF report

## Figures and Tables

**Table 1:** Hydrogen-bond geometry (Å, °) *Cg*1 is the centroid of the phenyl ring.

*D*—H⋯*A*	*D*—H	H⋯*A*	*D*⋯*A*	*D*—H⋯*A*
C4—H4*A*⋯*Cg*1^i^	0.99	2.74	3.577 (2)	143

Symmetry code: (i) 
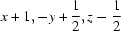
.

**Fig. 1.**
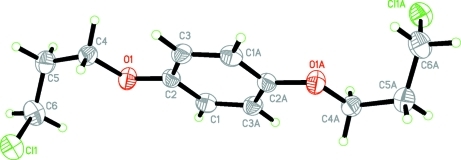
The molecular structure of the title molecule, with the atom-numbering scheme, symmetry code: -x+1, -y, -z+1. Displacement ellipsoids are drawn at the 75% probability level.

**Fig. 2.**
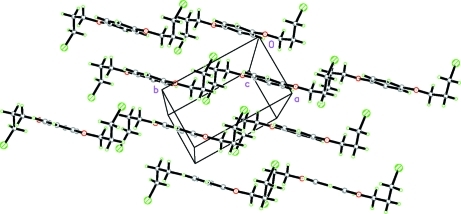
Part of the crystal structure of the title compound showing molecules being stacked along the c-axis.

## supplementary crystallographic information

### Comment

Alkoxybenzene derivatives are useful intermediates in organic synthesis (Dudones & Pearson, 2000; Chen *et al.*, 1996). Especially, halogenoalkoxybenzenes are used to synthesize diketopyrrolopyrrole derivatives which are a class of strongly fluorescent heterocyclic pigments and their structures could be easily optimized through variations of substituents at the 2,5- and 3,6-positions (Jin *et al.*, 2010; Rabindranath *et al.*, 2006; Zhang *et al.*, 2008; Zhu *et al.*, 2007). In this paper, the structure of the title compound synthesized, (I), and we report herein its crystal structure. In the molecule of (I) (Fig. 1) the bond lengths and angles are within normal ranges (Allen *et al.*, 1987). The asymmetric unit of the title compound contains a half of the molecule situated on a two-fold rotational axis. Intermolecular C4-H4···Cg1 interactions (Cg1 is the centroid of the phenyl ring ring) stabilize the crystal structure.

### Experimental

For the preparation of the title compound, p-dihydroxybenzene (11.0 g, 0.1 mol) was dissolved in dry acetone (100 ml). 1-Bromo-3-chloropropane (31.5 g, 0.2 mol) and potassium carbonate (138 g, 1mol) were added to this solution, the reaction was stirred under reflux for 11 h, The reaction mixture was filtered, the filtrate was concentrated, then washed with sodium hydroxide solution and extracted with ethyl acetate. After concentration, the residue was purified by recrystallization from chloroform (yield; 20.1 g, 76%, m.p. 339 K). Spectroscopic analysis: IR (KBr, ν, cm^-1^): 3085, 2957, 1541, 1351, 1032, 814. ^1^HNMR (400 MHz, CDCl_3_, ppm): 2.21 (m, 4H), 3.75 (t, 4H), 4.06 (t, 4H), 6.84 (d, 4H).

### Refinement

All H atoms were positioned geometrically and refined as riding (C-H = 0.95-0.99Å) and allowed to ride on their parent atoms, with U_iso_(H) =1.2U_eq_(parent).

### Crystal data

**Table:**

C_12_H_16_Cl_2_O_2_	*F*(000) = 276
*M**_r_* = 263.15	*D*_x_ = 1.383 Mg m^−^^3^
Monoclinic, *P*2_1_/*c*	Melting point: 339 K
Hall symbol: -P 2ybc	Mo *K*α radiation, λ = 0.71075 Å
*a* = 4.9813 (8) Å	Cell parameters from 2060 reflections
*b* = 8.3200 (14) Å	θ = 2.7–27.9°
*c* = 15.273 (2) Å	µ = 0.50 mm^−^^1^
β = 93.156 (6)°	*T* = 113 K
*V* = 632.02 (17) Å^3^	Prism, colorless
*Z* = 2	0.22 × 0.20 × 0.18 mm

### Data collection

**Table:**

Rigaku Saturn diffractometer	1502 independent reflections
Radiation source: rotating anode	1273 reflections with *I* > 2σ(*I*)
multilayer	*R*_int_ = 0.033
Detector resolution: 14.222 pixels mm^-1^	θ_max_ = 27.9°, θ_min_ = 2.7°
ω scans	*h* = −4→6
Absorption correction: multi-scan (*CrystalClear*; Rigaku, 2000)	*k* = −10→9
*T*_min_ = 0.899, *T*_max_ = 0.916	*l* = −20→20
5847 measured reflections	

### Refinement

**Table:**

Refinement on *F*^2^	Primary atom site location: structure-invariant direct methods
Least-squares matrix: full	Secondary atom site location: difference Fourier map
*R*[*F*^2^ > 2σ(*F*^2^)] = 0.031	Hydrogen site location: inferred from neighbouring sites
*wR*(*F*^2^) = 0.078	H-atom parameters constrained
*S* = 1.07	*w* = 1/[σ^2^(*F*_o_^2^) + (0.0407*P*)^2^ + ] where *P* = (*F*_o_^2^ + 2*F*_c_^2^)/3
1502 reflections	(Δ/σ)_max_ < 0.001
73 parameters	Δρ_max_ = 0.37 e Å^−^^3^
0 restraints	Δρ_min_ = −0.16 e Å^−^^3^

### Special details

**Table:**

Geometry. All e.s.d.'s (except the e.s.d. in the dihedral angle between two l.s. planes) are estimated using the full covariance matrix. The cell e.s.d.'s are taken into account individually in the estimation of e.s.d.'s in distances, angles and torsion angles; correlations between e.s.d.'s in cell parameters are only used when they are defined by crystal symmetry. An approximate (isotropic) treatment of cell e.s.d.'s is used for estimating e.s.d.'s involving l.s. planes.
Refinement. Refinement of *F*^2^ against ALL reflections. The weighted *R*-factor *wR* and goodness of fit *S* are based on *F*^2^, conventional *R*-factors *R* are based on *F*, with *F* set to zero for negative *F*^2^. The threshold expression of *F*^2^ > σ(*F*^2^) is used only for calculating *R*-factors(gt) *etc*. and is not relevant to the choice of reflections for refinement. *R*-factors based on *F*^2^ are statistically about twice as large as those based on *F*, and *R*- factors based on ALL data will be even larger.

### Fractional atomic coordinates and isotropic or equivalent isotropic displacement parameters (Å^2^)

**Table:**

	*x*	*y*	*z*	*U*_iso_*/*U*_eq_	
Cl1	0.22691 (7)	0.61216 (4)	0.66618 (2)	0.02618 (13)	
O1	0.16835 (18)	0.17363 (11)	0.60468 (6)	0.0199 (2)	
C1	0.5063 (2)	−0.01858 (15)	0.59005 (8)	0.0186 (3)	
H1	0.5102	−0.0314	0.6519	0.022*	
C2	0.3281 (3)	0.09041 (14)	0.54960 (8)	0.0167 (3)	
C3	0.3213 (3)	0.10898 (15)	0.45872 (9)	0.0185 (3)	
H3	0.1997	0.1829	0.4304	0.022*	
C4	−0.0045 (3)	0.29417 (15)	0.56500 (9)	0.0194 (3)	
H4A	−0.1349	0.2444	0.5218	0.023*	
H4B	0.1027	0.3745	0.5342	0.023*	
C5	−0.1508 (3)	0.37389 (16)	0.63743 (9)	0.0226 (3)	
H5A	−0.2664	0.2930	0.6644	0.027*	
H5B	−0.2697	0.4587	0.6114	0.027*	
C6	0.0331 (3)	0.44827 (16)	0.70864 (9)	0.0250 (3)	
H6A	0.1569	0.3651	0.7337	0.030*	
H6B	−0.0759	0.4887	0.7562	0.030*	

### Atomic displacement parameters (Å^2^)

**Table:**

	*U*^11^	*U*^22^	*U*^33^	*U*^12^	*U*^13^	*U*^23^
Cl1	0.0298 (2)	0.0224 (2)	0.0265 (2)	−0.00092 (14)	0.00272 (15)	−0.00187 (14)
O1	0.0221 (5)	0.0210 (5)	0.0167 (5)	0.0060 (4)	0.0016 (4)	0.0003 (4)
C1	0.0212 (7)	0.0210 (7)	0.0135 (6)	−0.0013 (5)	0.0005 (5)	0.0011 (5)
C2	0.0159 (7)	0.0165 (6)	0.0178 (7)	−0.0012 (5)	0.0015 (5)	−0.0018 (5)
C3	0.0184 (7)	0.0174 (6)	0.0193 (7)	0.0010 (5)	−0.0021 (5)	0.0016 (5)
C4	0.0199 (7)	0.0191 (6)	0.0190 (7)	0.0029 (5)	−0.0014 (5)	0.0003 (5)
C5	0.0224 (7)	0.0224 (7)	0.0234 (8)	0.0019 (5)	0.0043 (6)	0.0004 (6)
C6	0.0318 (8)	0.0227 (7)	0.0210 (7)	−0.0005 (6)	0.0056 (6)	−0.0005 (6)

### Geometric parameters (Å, °)

**Table:**

Cl1—C6	1.8113 (14)	C4—C5	1.5111 (17)
O1—C2	1.3762 (14)	C4—H4A	0.9900
O1—C4	1.4342 (15)	C4—H4B	0.9900
C1—C3^i^	1.3887 (17)	C5—C6	1.5151 (19)
C1—C2	1.3897 (17)	C5—H5A	0.9900
C1—H1	0.9500	C5—H5B	0.9900
C2—C3	1.3951 (18)	C6—H6A	0.9900
C3—C1^i^	1.3886 (17)	C6—H6B	0.9900
C3—H3	0.9500		
			
C2—O1—C4	116.58 (10)	C5—C4—H4B	110.2
C3^i^—C1—C2	120.91 (12)	H4A—C4—H4B	108.5
C3^i^—C1—H1	119.5	C4—C5—C6	114.07 (11)
C2—C1—H1	119.5	C4—C5—H5A	108.7
O1—C2—C1	115.67 (11)	C6—C5—H5A	108.7
O1—C2—C3	124.69 (12)	C4—C5—H5B	108.7
C1—C2—C3	119.63 (11)	C6—C5—H5B	108.7
C1^i^—C3—C2	119.46 (12)	H5A—C5—H5B	107.6
C1^i^—C3—H3	120.3	C5—C6—Cl1	111.33 (9)
C2—C3—H3	120.3	C5—C6—H6A	109.4
O1—C4—C5	107.47 (11)	Cl1—C6—H6A	109.4
O1—C4—H4A	110.2	C5—C6—H6B	109.4
C5—C4—H4A	110.2	Cl1—C6—H6B	109.4
O1—C4—H4B	110.2	H6A—C6—H6B	108.0
			
C4—O1—C2—C1	176.07 (11)	C1—C2—C3—C1^i^	−0.2 (2)
C4—O1—C2—C3	−3.86 (18)	C2—O1—C4—C5	−177.69 (10)
C3^i^—C1—C2—O1	−179.68 (10)	O1—C4—C5—C6	57.71 (14)
C3^i^—C1—C2—C3	0.2 (2)	C4—C5—C6—Cl1	64.36 (13)
O1—C2—C3—C1^i^	179.68 (11)		

Symmetry codes: (i) −*x*+1, −*y*, −*z*+1.

### Hydrogen-bond geometry (Å, °)

**Table:**

Cg1 is the centroid of the phenyl ring.

**Table:**

*D*—H···*A*	*D*—H	H···*A*	*D*···*A*	*D*—H···*A*
C4—H4A···Cg1^ii^	0.99	2.74	3.577 (2)	143.

Symmetry codes: (ii) *x*+1, −*y*+1/2, *z*−1/2.
